# Gender norms about romantic relationships and sexual experiences among very young male adolescents in Korogocho slum in Kenya

**DOI:** 10.1007/s00038-020-01364-9

**Published:** 2020-04-09

**Authors:** Beatrice W. Maina, Benedict O. Orindi, Yandisa Sikweyiya, Caroline W. Kabiru

**Affiliations:** 1 School of Public Health, University of the Witwatersrand, Johannesburg, South Africa; 2 African Population and Health Research Center, APHRC Campus, Manga Close, Off Kirawa Road, P.O. Box 10787-00100, Nairobi, Kenya; 3 Gender and Health Research Unit, South African Medical Research Council, Pretoria, South Africa; 4 Population Council, Nairobi, Kenya

**Keywords:** Early male adolescents, Sexual behaviors, Gendered norms, Urban informal settlements, Kenya

## Abstract

**Objectives:**

To investigate the association between gender norms about romantic relationships and sexual experiences of very young male adolescents (VYMA) living in Korogocho slum in Nairobi, Kenya.

**Methods:**

We used cross-sectional data from a sample of 426 VYMA living in Korogocho slum. We conducted an exploratory factor analysis and confirmatory factor analysis to, respectively, explore and validate the factor structure underlying gender norms scale items. We used structural equation modelling to assess the association between gender norms and sexual experiences of VYMA.

**Results:**

We found high endorsement of heteronormative beliefs about romantic relationships and low endorsement of sexual double standards. Sexual experience was associated with low endorsement of heteronormative beliefs, being pre-pubertal, school absenteeism and being below recommended grade for age. Sharing a sleeping room with more than two people, been born outside Nairobi, and living in households headed by older persons lowered the likelihood of sexual experience.

**Conclusions:**

Our findings underscore the need for further research to understand how gender norms evolve as young boys transition through adolescence to adulthood and how these changes impact on sexual behaviors

**Electronic supplementary material:**

The online version of this article (10.1007/s00038-020-01364-9) contains supplementary material, which is available to authorized users.

## Introduction

Gender norms, which are widely understood to refer to social expectations of appropriate roles and behaviors for males and females (Ratele 2015; Ridgeway and Correll 2004; Ryle 2011), are increasingly being recognized as drivers of health and well-being. The scripts for appropriate behavior based on one’s sex are informed by collective beliefs about what most people do (descriptive norms), perceptions of what people ought to do (injunctive norms) and observing what popular people do (cohesive norms) (Addis and Mahalik 2003). While there are a number of factors contributing to gender inequalities (Kågesten et al. 2016), gender theorists show that stereotypical gender norms promote inequalities between men and women (Connell and Messerschmidt 2005; Ridgeway and Correll 2004; Ryle 2011). These gender inequalities negatively influence sexual and reproductive health behaviors resulting in sexual coercion and abuse, intimate partner violence, unplanned pregnancies, sexually transmitted infections (STIs) and HIV (Firestone et al. 2003; Nyamhanga and Frumence 2014), issues that impact on an individual’s well-being across the life course.

While gendered norms are learned and enacted throughout the life course (Ridgeway and Correll 2004; Ryle 2011), it is in early adolescence—the period between 10 and 14 years—that intensification of gender learning takes place (Hill and Lynch 1983; Pettitt 2004). The intensification of gender learning is attributed to developmental changes taking place in early adolescence, including pubertal maturation and interest in romantic relationships (Pettitt 2004). It is in this period that gendered behaviors in romantic relationships begin to emerge. Gender norms vary across social and cultural contexts (Nyamhanga and Frumence 2014; Shefer et al. 2015); hence, norms within contexts in which young adolescents live are likely to influence how they behave as sexual beings (Izugbara and Undie 2008; Shefer et al. 2015).

Whereas extensive research has been conducted on how gender norms affect girls’ and women’s health (Firestone et al. 2003; Nyamhanga and Frumence 2014; Ratele 2015), there is little, but growing, focus on the interrelationship between gender norms and men’s sexual health (Addis and Mahalik 2003; Ratele 2015; Shefer et al. 2015), especially among early adolescents (10–14 years), also referred to as very young male adolescents (VYMA) (De Meyer et al. 2017; Gevers et al. 2013; Kågesten et al. 2016; Ray et al. 2012). A systematic review on gender attitudes in early adolescence found that boys endorse gender norms that promote inequalities between men and women (Kågesten et al. 2016). The review also recognizes the scarcity of data on gender norms in early adolescence noting that majority of studies reviewed were from North America and Western Europe (Kågesten et al. 2016).

Extant literature posits sexual development as a normative continuum from friendship to romantic relationships and ultimately to penetrative sexual relationships (Gevers et al. 2013; Tolman and McClelland 2011). It is in early adolescence that sexual development intensifies as young people experience pubertal changes (Galambos et al. 1990). Sexual behaviors in early adolescence are sporadic and unplanned and include normative and risky behaviors (Hensel et al. 2008). With some exceptions (Kabiru et al. 2010; Kagesten et al. 2018), most studies on sexual behaviors in sub-Saharan Africa have typically overlooked non-penetrative forms of sexual behaviors that are important markers of sexual experience (Folayan et al. 2015; Marston et al. 2013; McGrath et al. 2009), and thus, little evidence on the prevalence of such behaviors among early adolescent boys exist.

Social and cultural expectations may prevent men from protecting themselves from sexual risks without jeopardizing their masculine identities (Izugbara and Undie 2008; Ratele 2015; Shefer et al. 2015). It is therefore important to understand, at an early age, how gender norms may affect males’ sexual health and behaviors. This study investigates the association between endorsement of gender norms about romantic relationships and sexual experiences of VYMA living in Korogocho slum in Nairobi, Kenya’s capital city. Acknowledging the wide range of sexual experiences in early adolescence (Kabiru et al. 2010; Kagesten et al. 2018), we include both penetrative and non-penetrative sexual experiences as markers of sexual development.

## Methods

### Study design and setting

This study used cross-sectional data collected in December 2018 from 426 VYMA aged 10–14 years living in households covered by the Nairobi Urban Health and Demographic Surveillance System (NUHDSS) in Korogocho informal settlement. Korogocho is one of the most congested slums in Nairobi with a population of about 200,000 in an area of 0.5 km^2^ (Beguy et al. 2015). The slum is characterized by high levels of crime, social and physical insecurity, unemployment, overcrowding and limited access to health (Beguy et al. 2015). The NUHDSS provided an accessible sampling framework for this study.

### Study population

The June 2018 NUHDSS enumeration list was used as a sampling frame to identify boys aged 10–14 years at the time of data collection. From the eligible 1878 boys, a random sample of 550 boys, stratified by age, was drawn, of whom 426 (78%) were successfully interviewed. Of the 124 boys who were not reached, about half (*n* = 65) had moved out of the surveillance area, 26 were away for an extended period or their structures had been demolished, and 25 could be not traced. Two boys had died since the NUHDSS enumeration listing was done, one boy refused to be interviewed and two parents refused to have their child interviewed. Data from three boys were excluded from the analysis as they did not respond to the sexual behavior questions.

### Data collection procedures

Data collection tools used in this study were developed during phase 1 of the Global Early Adolescent Study (GEAS). The GEAS is the first international study to focus on gender norms and sexuality among very young adolescents (Chandra-Mouli et al. 2017). The questionnaires covered information related to the individual, family, peers, school, neighborhood, media, adolescent physical and mental health, sexual behaviors, empowerment, future aspirations and gender norms about romantic relationships in early adolescence. Additional questions on household characteristics were administered to parents/guardians of participating boys. Data were collected electronically using android tablets loaded with Survey CTO v12.2. Seventeen trained male interviewers administered the questionnaire using computer-assisted personal interview.

The process of data collection was structured. First, we identified households of sampled adolescents using the NUHDSS listing information. A parent/guardian in the household was approached, provided with information about the study and requested for permission to allow the sampled adolescent to participate in the study and also for the parent/guardian to be interviewed on household characteristics. If they gave permission, they were requested to provide written informed consent for their own participation and for their son/male ward to be interviewed. A household characteristics questionnaire was then administered to the parent/guardian, after which a card with a unique number and NUHDSS identification details were given. This card was proof that the parent/guardian had given informed consent for the adolescent to participate in the study and they had been interviewed about their household characteristics. The sampled adolescent was required to bring this card to the interview site and, if they assented to participate in the study, they would be interviewed. All the cards were collected after the adolescent interview. To provide a safe environment for conducting interviews and to ensure privacy and confidentiality during the interview process, all adolescent interviews were conducted at a local school within the NUHDSS site.

### Measures

#### Outcome variables

The outcome of interest is sexual experience, conceptualized as having engaged in penetrative and/or non-penetrative sexual activities. Penetrative activities for the purpose of this study comprise penile-vaginal or penile-anal sexual intercourse while non-penetrative activities include being in love, spending time alone, holding hands, kissing, hugging/cuddling, touching, flirting over the phone, sharing sexual pictures of themselves and oral sex. While sexual intercourse is often used as the main marker of sexual experience (Gevers et al. 2013; Marston et al. 2013), several studies show that young people engage in other forms of non-penetrative sexual behaviors such as kissing, spending time alone and cuddling among others, in a continuum or concurrently with sexual intercourse (Bankole et al. 2007; Kabiru et al. 2010; Kagesten et al. 2018). A significant proportion of adolescents engaging in non-penetrative sexual behaviors may not have engaged in any penetrative behaviors (Kabiru et al. 2010).

Sexual experience questions were structured such that they only captured experiences in romantic/sexual contexts. We first introduced the sexual behavior module as follows “Now we would like to ask you about things that YOU might have done together with someone else as more than just friends (i.e. with a boyfriend/girlfriend)”. This was followed by questions referring to sexual experiences such as (1) Have you ever spent time alone with someone you were in love with in a private space without any adults around? (2) Have you ever held hands with someone you were in love with? As sexual experiences among young people are sporadic (Bankole et al. 2007; Hensel et al. 2008), lifetime engagement (ever) rather than current (engaged in sexual activity in the last 12 months) was used.

#### Explanatory variables

The main explanatory variable was the multidimensional gender norms scale, comprising 46 items measuring individual beliefs about gender norms. Responses to the items were provided on a 5-point Likert scale ranging from “agree a lot” (= 1) to “disagree a lot” (= 5). In addition, the questionnaire had a code for “don’t know” and “refused to answer” responses. In the present analysis, don’t know responses were combined with the neutral group of “neither agree nor disagree” while “refused to answer” responses were treated as missing data. Missing data ranged from 0 to 1% per gender norm item. Using factor analysis, we extracted four factors from the gender norms scales items corresponding to four domains of sexual double standards, heteronormative sexual relationships, stereotypical views about toughness and weakness in relationships and relational autonomy (see supplementary material). The scale’s overall reliability was high (Cronbach's alpha = 0.79); however, two factors namely “relational autonomy” and “stereotypical views about toughness” had an internal consistency lower than 0.6 (0.543 and 0.485, respectively) and were not included in subsequent analyses.

Data on a range of exogenous variables, which based on the existing literature (Bankole et al. 2007; Houck et al. 2012; Kagesten et al. 2018) are associated with adolescent sexual behavior, were collected. They included age, ethnicity, religion, place of birth, pubertal maturation (which was self-assessed through questions about onset of genital growth, hair growth and/or voice change for boys), household size, number of rooms in the house, sleeping arrangements, parental survivorship, age of the household head, grade for age and school absenteeism during the past month. Two exogenous variables (grade for age and school absenteeism during the past month) had missing values for two boys who were out of school at the time of the study.

### Statistical analysis

All descriptive analyses were performed using STATA version 15 (StataCorp, College Station, TX), and all regressions were performed using Mplus version 7.4 (Muthén and Muthén 1998–2015). Having determined the factor structure of the gender norms scale (see supplementary material), we calculated a mean score on each of the factors (subscales) such that higher scores indicated greater endorsement of gendered attitudes in romantic relationships and compared these mean scores between those reporting engagement in sexual activities and those who did not. Within the structural equation modelling (SEM) framework [see, for example, Kline (2015)], the sexual experiences outcome was regressed on the gender norms factors as well as on the selected exogenous variables. Exogenous variables to be adjusted for in the SEM model were selected based on univariate multiple probit model: exogenous variables found to be significant at *p* ≤ 0.1 in the univariate multiple model (data not shown) were included in the SEM model. Age was included even if it was not significant as we wished to adjust for its effect. As such seven exogenous variables; age, place of birth, pubertal maturation, sleeping arrangements, age of household head, grade for age and school absenteeism were adjusted for in the SEM model. Confirmatory factor analysis (CFA) and SEM models were identified by setting the scales of the factors equal to one; and standardized estimates were requested.

For both the CFA and SEM, a probit link function was assumed as it is also the default in Mplus for categorical data. Similar to CFA, the SEM model fit was evaluated using the comparative fit index (CFI), Tucker–Lewis index (TLI), root mean squared error of approximation (RMSEA) and standardized root mean square residual (SRMR). A reasonably good fitting model is obtained when SRMR is ≤ 0.08, RMSEA is ≤ 0.06, and CFI and TLI are ≥ 0.95. A model with CFI and TLI values of at least 0.90 is deemed acceptable (Hu and Bentler 1999). We also report here that the factor structure of the gender norms scale reported in Table S2 was not distorted by including structural relations in the model.

## Results

### Descriptive findings

Table 1 shows the sexual experiences of the participants. In total, 24% of boys had ever been in love, 19% had ever been in a romantic relationship, and 22% reported at least one sexual experience. About 7% of boys reported sexual intercourse. Among those who reported sexual experience (*n* = 93), experiences ranged from spending time alone with someone they were in love with (90%) to anal sex (3%).

**Table 1:** Sexual and romantic experiences among very young male adolescents in Korogocho slums, Kenya 2018

Characteristic	% (col)	*N* [Unweighted]
Ever been in love (*N* = 426)	23.9	102
Ever been in a relationship (*N* = 426)	19.3	82
*Relationship status *(*n* = 82)		
In a current relationship	42.7	35
In a past relationship	57.3	47
Sexual experience (*N* = 426)	21.8	93
*Type of sexual experience *(*n* = 93)		
Spent time alone	96.8	90
Held hands	46.2	43
Hugged/cuddled	35.5	33
Kissed	11.8	11
Flirted/shared sexual picture	5.4	5
Touched/fondled private parts	26.9	25
Sexual intercourse	31.2	29
Oral sex	5.4	5
Anal sex	3.2	3

Table 2 presents boys’ sexual experience by their background characteristics. The proportion of boys reporting any sexual activity varied by age, ethnicity, religion (denomination), pubertal maturation, sleeping arrangements, grade for age and school absenteeism, factors that were all found to be significantly associated with sexual experience at bivariate level. Place of birth and age of the household head were found to be marginally significant (90% confidence interval).

**Table 2:** Proportions of very young male adolescents living in Korogocho slums by background characteristics, Kenya 2018

Characteristic	Total (*N*)	Number ever had sexual experience	Percent (row) ever had sexual experience	*P *value
Age				0.019
10–12	260	47	18.1	
13–14	166	46	27.7	
Ethnicity				0.026
Kikuyu	135	35	25.9	
Luo	107	30	28.0	
Luhya	64	14	21.9	
Somali/Garre/Borana/Gabra/Banna	90	10	11.1	
Others	30	4	13.3	
Religion (denomination)				0.007
Christian	307	76	24.8	
Muslim	96	10	10.4	
None	23	7	30.4	
Place of birth				0.085
Nairobi	315	75	23.8	
Outside Nairobi	111	18	16.2	
Pubertal maturation				< 0.001
Started puberty	197	58	29.4	
Not started puberty	229	35	15.3	
Household size				0.336
2–5 persons	234	47	20.1	
5 persons and above	192	46	24.0	
Household rooms				0.123
One room	182	35	19.2	
Two rooms	177	47	26.6	
Three or more rooms	67	11	16.4	
Sleeping arrangements				< 0.001
1–2 people	71	26	36.6	
3–4 people	184	26	14.1	
5 or more people	171	41	24.0	
Parental survivorship				0.704
Both parents alive	367	79	21.5	
One/both parents dead	59	14	23.7	
Age of household head				0.080
Below 50 years	241	60	24.9	
50 years and above	185	33	17.8	
Grade for age				0.041
Recommended grade or above	283	53	18.7	
Below recommended grade	141	39	27.7	
School absenteeism				0.037
Never missed school	233	42	18.0	
Missed school one or more times	191	50	26.2	

### Endorsement of gender norms about romantic relationships

As shown in Table 3, there was low endorsement to the sexual double standard scale, with no major differences between those with and without sexual experience. Low endorsement of sexual double standard score means that boys did not agree with the conventional belief that girls and boys should be judged differently relative to the same sexual behavior. While there was overall high endorsement of heteronormativity in romantic relations, boys who reported no sexual activity were more likely to endorse heteronormativity in romantic relationships than those reporting any sexual activity. High endorsement of heteronormative romantic relationships implies that boys agreed with beliefs of male dominance and passivity of females in romantic relationships.

**Table 3:** Endorsement of gender norms about romantic relationships by sexual experience among very young male adolescents in Korogocho slums, Kenya 2018

Gender norms domains	Ever had sexual experience	Never had sexual experience
Sexual double standard (mean score)	2.3	2.4
Heteronormative romantic relationships (mean score)	3.1	3.9

### Associations between sexual experiences and gender norms

Table 4 shows the associations between the gender norms factors and VYMA’s sexual experiences, after adjusting for age of the boys, place of birth, pubertal maturation, sleeping arrangements, age of household head, grade for age and school absenteeism using SEM. The heteronormative romantic relationships subscale had a significant inverse association with sexual experiences meaning that boys who endorsed norms of male dominance in romantic relationships were less likely to have had a sexual experience. The sexual double standards subscale was not significantly associated with sexual experience.

**Table 4:** Association between gender norms endorsement domains and sexual behavior of very young male adolescents in Korogocho slums, Kenya 2018

Parameter	Sexual experience		
	Estimate	Standard error	*P *value
*Gender norms factors*			
Sexual double standard	0.045	0.065	0.490
Normative romantic relationships	− 0.384	0.063	< 0.001
*Age*			
10–12	Ref		
13–14	0.128	0.154	0.406
*Place of birth*			
Nairobi	Ref		
Outside Nairobi	− 0.288	0.145	0.047
Pubertal maturation			
Started puberty	Ref		
Not started puberty	0.330	0.146	0.024
*Sleeping arrangements*			
1–2 people	Ref		
3–4 people	− 0.644	0.176	< 0.001
5 or more people	− 0.299	0.177	0.091
*Age of household head*			
Below 50 years	Ref		
50 years and above	− 0.295	0.143	0.039
*Grade for age*			
Recommended grade or above	Ref		
Below recommended grade	0.296	0.135	0.028
*School absenteeism (past month)*			
Never missed school	Ref		
Ever missed school	0.348	0.139	0.012

Boys who had not experienced any pubertal changes, those below the recommended grade for age and those who had missed school at any time during the 6 months preceding the survey were more likely to report sexual activity. On the other hand, being born outside Nairobi, sleeping in a room with two or more people and living in a household where the head was aged 50 years or older was associated with a lower likelihood of reporting any sexual activity. The SEM model provided an acceptable fit to the data [fit statistics: CFI = 0.90; TLI = 0.90, RMSEA = 0.05 (0.04–0.06), WRMR = 1.34] (Table S3).

## Discussion

The current study examined the association between gender norms and sexual experiences among VYMA aged 10–14 years living in Korogocho slums in Nairobi, Kenya. Firstly, we examined sexual experiences among VYMA and found that about one in five boys reported penetrative or non-penetrative sexual experiences with the majority reporting non-penetrative sexual activities. This finding suggests that young people begin being romantically involved at an early age, confirming findings from past studies globally (Bankole et al. 2007; Kagesten et al. 2018) showing that young people may engage in a diverse range of intimate or romantic activities, even if they report that they have never had penetrative sexual intercourse. Early involvement in intimate or romantic activities may increase the likelihood of sexual risk behaviors that could ultimately lead to poor health outcomes (Kagesten et al. 2018; Kugler et al. 2017; Shefer et al. 2015). For instance, a study conducted among male and female students in 7th–12th grades in the USA found that early sexual intercourse predicted having more than one partners in the year preceding the study (Kugler et al. 2017).

Secondly, we examined the extent to which VYMA endorsed gendered beliefs about romantic relationships and examined the association between the gender constructs and sexual behavior. Our findings showed two constructs that were relevant to this study namely sexual double standards and heteronormative romantic relationships. Sexual double standards measured the extent to which boys endorsed beliefs that boys and girls should be judged differently relative to the same sexual behaviors, while heteronormative romantic relationships measured the belief about male dominance and passivity of females in romantic relationships. While low endorsement of sexual double standards resonates with evolving social expectations towards sexual permissiveness (Xiayun et al. 2012), high endorsement of heteronormative romantic relationships shows that social norms governing how men and women relate in romantic relationships exist and is a portrayal of gender dynamics and power imbalances in romantic and sexual relationships (De Meyer et al. 2017).

The inverse relationship between endorsement of heteronormative romantic relationships and sexual experiences at multivariate level even when we controlled for exogenous variables is important for adolescent research. This finding contradicts the past research suggesting that traditional beliefs about male dominance in romantic relationships are associated with early sexual debut (Ampofo 2001) and could be interpreted in different ways. First, while VYMA exhibit an understanding of social expectations about romantic relationships and the power dynamics in heterosexual relationships, they are yet to project these beliefs into their sexual activities. Secondly, with majority of gender equality programs targeting girls and women (Birdthistle et al. 2018; Kågesten et al. 2016), it is probable that girls are empowered to make informed and autonomous decisions about their sexual activities, and thus diminishing male dominance in sexual relationships. As such, as they are involved in sexual and romantic relationships, boys may reassess gender norms and adapt their values and personal beliefs.

The finding that boys who were born outside Nairobi, majority of whom were born in rural areas of Kenya, were less likely to report sexual activity is noteworthy. While we did not find any available evidence on differentials in sexual behaviors among adolescent boys in urban slums by migration status, studies comparing sexual behaviors of adolescent boys in rural and urban areas report mixed findings (Folayan et al. 2015; Ray et al. 2012). For instance, a study among rural and urban adolescents in India found that urban boys were less likely to be involved in penetrative sexual activity (Ray et al. 2012). On the other hand, a study conducted in rural and urban South Africa found that urban adolescents were more likely to have had a non-penetrative sexual experience compared to rural adolescents (Peltzer 2006). Though Peltzer (2006) combined data from both male and female adolescents, the findings are important in defining the differentials in sexual outcomes among adolescents. Rural–urban differentials in sexual socialization could explain our findings that boys born in urban informal settlements are likely to start engaging in sexual activity early (Folayan et al. 2015). Folayan et al. (2015) argue that availability and accessibility of sexually explicit media in urban settings may expose young people to social pressure to engage in sexual activity earlier than their rural counterparts. Moreover, the harsh conditions in urban slums in Kenya, which include poor housing and poverty, expose young people to sexual activity at an early age (De Meyer et al. 2017).

Our study also found that boys who had not started puberty were more likely to have had a sexual experience compared to those who reported pubertal changes. This finding suggests that some boys begin to engage in sexual activities before they reach puberty. Past findings on pubertal timing and sexual experiences of adolescent boys are mixed (Bello et al. 2017; Halpern et al. 1993). Using panel data, Halpern et al. (1993) argue that the association between pubertal changes and sexual experiences is not solely based on changes in hormonal levels but also due to social stimuli. Early exposure to sexual activity and content may exert pressure on VYMA to engage in sexual activity. De Meyer et al. (2017) found that adolescents in urban slums in Nairobi were exposed to sexual activity at an early age. Our findings underscore the need for programs to target pre-pubertal young people who live in contexts that expose them to sexual risks at an early age.

Boys who slept in a room with more than two people were less likely to have had a sexual experience. Dodoo et al. (2007) argue that when young people share a sleeping room with adults, they are likely to be socialized to early sexual activity due to lack of the privacy necessary for the adults to engage in sexual activity. Despite the early socialization, we also argue that sharing a room with more people does not offer young boys the privacy needed to engage in sexual activity within the household. The finding that living in households headed by older persons (> 50 years) was associated with a lower likelihood of engaging in sexual activity may point to differentials in parental monitoring and intergenerational gaps in the socialization processes. Parental monitoring and supervision have been found to be an important influence on adolescents’ sexual behaviors (Telzer et al. 2018), and monitoring and supervision styles are likely to differ between older and younger parents. While we did not find the existing literature on association between age of household head and sexual experiences among adolescent boys, Izugbara (2015) found that girls living in households with older adult heads were less likely to report a pregnancy.

Boys who were below their recommended grade for age and those who had missed school had a higher likelihood of engaging in sexual activity. The literature suggests that schooling is a protective factor and is associated with later sex debut among boys (McGrath et al. 2009). School absenteeism may provide young boys with unsupervised time away from school, where opportunities to engage in sexual activity could arise. Being below recommended grade for age is often seen as an indicator for poor academic performance. Our findings that being below recommended grade for age was associated with a higher likelihood for sexual experience confirm past findings that young people who are doing well in school are less likely to engage in risky sexual behavior (Houck et al. 2012).

Our findings should be interpreted in the light of the following limitations. First, this study focused on VYMA living in an informal urban settlement, and thus, generalization of these findings is limited to populations living in similar settings. Second, this study used cross-sectional data, and so, causal relationships between endorsement of heteronormative beliefs and sexual experiences cannot be determined. Third, measures used in this study were self-reported, with a possibility of social desirability bias. While data collectors were well trained to conduct interviews ensuring utmost privacy and confidentiality, there still existed potential to under-report or over-report some of the measures, especially on gender beliefs and sexual experiences.

### Conclusion

The inverse association between endorsement of heterosexual gender beliefs and sexual activity highlights the need for further research to understand how gender norms evolve as young boys transition through adolescence to adulthood and how these changes impact on sexual behaviors in order to inform programming particularly in settings like urban slums where young people may be at high risk for early sexual debut. Early adolescence presents a window of opportunity for early interventions to promote gender-equitable norms and health sexual behaviors.

## Electronic supplementary material

Below is the link to the electronic supplementary material.

Supplementary file1 (DOCX 39 kb)
