# Correction to: Gender norms about romantic relationships and sexual experiences among very young male adolescents in Korogocho slum in Kenya

**DOI:** 10.1007/s00038-020-01512-1

**Published:** 2020-11-03

**Authors:** Beatrice W. Maina, Benedict O. Orindi, Yandisa Sikweyiya, Caroline W. Kabiru

**Affiliations:** 1 School of Public Health, University of the Witwatersrand, Johannesburg, South Africa; 2 African Population and Health Research Center, APHRC Campus, Manga Close, Off Kirawa Road, P.O. Box 10787-00100, Nairobi, Kenya; 3 Gender and Health Research Unit, South African Medical Research Council, Pretoria, South Africa; 4 Population Council, Nairobi, Kenya

## Correction to: International Journal of Public Health (2020) 65:497–506 10.1007/s00038-020-01364-9

The authors would like to correct an error in the publication of the original article. The error is described below and correct details provided. What is presented in the lines 23, 29, 38 , and in table 4 is the corrected version.

## Error

On page 503, Table 4, the labels on pubertal maturation variables were swapped. This resulted in an erroneous interpretation of the finding on page 502 and discussion of the finding on 503 and 504 (Table 4).

## Correction

**Table 4:** Association between gender norms endorsement domains and sexual behavior of very young male adolescents in Korogocho slums, Kenya 2018

Parameter	Sexual experience		
	Estimate	Standard error	*P* value
*Gender norms factors*			
Sexual double standard	0.045	0.065	0.490
Normative romantic relationships	− 0.384	0.063	< 0.001
*Age*			
10–12	Ref		
13–14	0.128	0.154	0.406
*Place of birth*			
Nairobi	Ref		
Outside Nairobi	− 0.288	0.145	0.047
*Pubertal maturation*			
Not started puberty	Ref		
Started puberty	0.330	0.146	0.024
*Sleeping arrangements*			
1–2 people	Ref		
3–4 people	− 0.644	0.176	< 0.001
5 or more people	− 0.299	0.177	0.091
*Age of household head*			
Below 50 years	Ref		
50 years and above	− 0.295	0.143	0.039
*Grade for age*			
Recommended grade or above	Ref		
Below recommended grade	0.296	0.135	0.028
*School absenteeism (past month)*			
Never missed school	Ref		
Ever missed school	0.348	0.139	0.012

## Results: Associations between sexual experiences and gender norms

Page 502: Second paragraph, first sentence

Boys who had experienced any pubertal changes, those below the recommended grade for age and those who had missed school at any time during the 6 months preceding the survey were more likely to report sexual activity.

## Discussion

Page 503, last paragraph extending to page 504

First sentence: Our study also found that boys who reported pubertal changes were more likely to have had a sexual experience compared to those who reported no pubertal changes suggesting that pubertal changes are associated with initiation of sexual activity. Sexual development is a major component of pubertal maturation (Fortenberry 2013; Kar et al. 2015).

Page 504, first paragraph (same paragraph as above), last sentence

Our findings underscore the need for programs to target young boys as they get into puberty especially those that live in contexts that expose them to sexual risks at an early age.
