# Supplementary material for: Gender norms about romantic relationships and sexual experiences among very young male adolescents in Korogocho slum in Kenya
Source: Int J Public Health. 2020 Apr 9;65(4):497–506. doi: 10.1007/s00038-020-01364-9 (PMC7275025; doi:10.1007/s00038-020-01364-9)
Supplement: Supplementary file 1 — Supplementary file1 (DOCX 39 kb) [file 38_2020_1364_MOESM1_ESM.docx]

**International Journal of Public Health**

**Gender norms about romantic relationships and sexual experiences among very young male adolescents in Korogocho slum in Kenya**

Beatrice W. Maina^1,2^, Benedict O. Orindi^2^, Yandisa Sikweyiya^1,3^, Caroline W. Kabiru^1,4^

**Affiliations**

1. School of Public Health, University of the Witwatersrand, Johannesburg, South Africa
2. African Population and Health Research Center, Nairobi, Kenya
3. South Africa Medical Research Council, Pretoria, South Africa
4. Population Council, Nairobi, Kenya

**Factorial structure of the gender norms scale**

For the gender norm scale, we first performed an exploratory factor analysis (EFA) to explore the factor structure underlying the 46 gender norms scale items. The EFA was likelihood-based using a robust weighted least squares estimation method with Geomin rotation. Factors were retained on the basis of the Kaiser’s ‘eigenvalue rule’ which suggests retaining eigenvalues larger than 1. In addition, we also observed the scree plot. A sharp drop in the scree plot signals that subsequent factors are ignorable as the amount of information in each successive factor is less than in its predecessors. Internal consistency of items on a factor was measured using Cronbach’s alpha coefficient. A minimum Cronbach’s alpha of 0.60 is considered adequate. A factor loading of ≥0.4 was considered salient. If an item loaded on more than one factor, it was considered to load on the factor with the higher standardized loading. Thus, item cross-loadings were fixed to zero, else the model would be non-identified resulting into computational problems. Items that did not load well on any of the identified factors were dropped. Next, a confirmatory factor analysis (CFA) was performed to validate the factor structure proposed by the EFA.

Seventeen items did not load well on any factor; that is, they had a factor loading <0.4 on all factors and were dropped from the analysis. Thus, the analyses were performed on 30 items. Supplementary Table S1 presents a complete list of items which were dropped from the analysis.

Using EFA, four factors were extracted, corresponding to four domains of sexual double standards, heteronormative sexual relationships, stereotypical views about toughness and weakness in relationships and relational autonomy. The four domains accounted for 49% of the variability in the 29 items. Model fit was evaluated using the comparative fit index (CFI), Tucker-Lewis index (TLI), root mean squared error of approximation (RMSEA), and standardized root mean square residual (SRMR). A reasonably good fitting model is obtained when SRMR is ≤0.08, RMSEA is ≤0.06, and CFI and TLI are ≥0.95. A model with CFI- and TLI-values of at least 0.90 are deemed acceptable. The 4-factor solution summarized the 29 items reasonably well (fit statistics: CFI= 0.967; TLI= 0.955, RMSEA= 0.057 (0.052-0.063), SRMR= 0.055; Table S2). The EFA solution is presented in supplementary Table S2.

Thirteen items (i.e., items 1, 3, 5, 7, 9, 11, 12, 14, 15, 17, 21, 23, 30) loaded on the ‘sexual double standard’ factor which measured the extent to which boys and girls were judged differently relative to the same sexual behaviors; 6 items (i.e., items 6, 8, 18, 20, 22, 27) loaded on the ‘heteronormative romantic relationships’ factor, measuring the beliefs that boys should play an active role and dominate romantic relationships while girls should be passive; 7 items (i.e., items 4, 10, 13, 16, 24, 25, 28) loaded on the ‘relational autonomy’ factor, a construct that measured the extent to which the belief that boys had more freedom in romantic relationships was endorsed; and 3 items (items 40 – 42) loaded on the ‘stereotypical views about toughness’ factor measuring the belief that male physical strength is important in romantic relationships

The CFA model results verified the hypothesized factor structure. Table S4 shows the standardized factor loadings and factor correlations. The scale’s overall reliability was high (Cronbach alpha=0.79), however two factors namely ‘relational autonomy’ and ‘stereotypical views about toughness’ had an internal consistency lower than 0.6 (0.543 for the ‘relational autonomy’ factor and 0.485 for the ‘stereotypical views about toughness’) and were not included in subsequent analysis. (Table S4).

**Table S1. Gender norm scale items that did not load well on any factor and were thus dropped from the analysis, Kenya 2018**

| **Gender norm scale items** |
| --- |
| 2. A boy and a girl your age should be able to spend time together alone if they want to |
| 19. Boys feel they should have girlfriends because their friends do |
| 26. In general, if an adolescent girl says “no” to sex her boyfriend will dump her |
| 29. It’s the girl’s responsibility to prevent pregnancy |
| 31. Girls should avoid playing sports with boys because they get hurt easily |
| 32. Boys should be raised tough so they can overcome any difficulty in life |
| 33. Girls should avoid raising their voice to be lady like |
| 34. Boys should always defend themselves even if it means fighting |
| 35. Girls are expected to be humble |
| 36. Girls should always fight back if boys try to take advantage of them |
| 37. Girls need their parents protection more than boys |
| 38. Boys should be able to show their feelings without fear of being teased |
| 39. Boys who behave like girls are considered weak |
| 43. Boys and girls should be equally responsible for household chores |
| 44. A woman’s role is taking care of her home and family |
| 45. A man should have the final word about decisions in the home |
| 46. A woman should obey her husband in all matters |

**Table S2. Exploratory factor analysis solution for the gender norms scale, Kenya 2018**

| **Items** | **Factors** | | | | | | |
| --- | --- | --- | --- | --- | --- | --- | --- |
|  | **F1** | | **F2** | | **F3** | | **F4** |
|  |  | |  | |  | |  |
| 1. A girl will lose interest in studying if she has a boyfriend | 0.520* | | -0.285* | | 0.014 | | 0.022 |
| 3. Girls your age often get into "trouble" when they have boyfriends | 0.619* | | -0.162* | | -0.154* | | 0.083 |
| 4. A boy should be able to have a girlfriend if he wants to | -0.042 | | 0.210* | | 0.554* | | 0.177* |
| 5. Boys have girlfriends for fun more than love | 0.446* | | 0.090 | | 0.081 | | 0.035 |
| 6. It’s normal for a boy your age to want a girlfriend | 0.114* | | 0.478* | | 0.200* | | 0.025 |
| 7. Girls who have boyfriends are irresponsible | 0.564* | | -0.353* | | 0.004 | | 0.058 |
| 8. Boys should have girlfriends to discover love | 0.012 | | 0.427* | | 0.349* | | 0.026 |
| 9. Boys like girls who wear revealing clothes | 0.471* | | 0.071 | | 0.099 | | -0.044 |
| 10. A girl should be able to have a boyfriend if she wants to | -0.023 | | 0.248* | | 0.549* | | 0.076 |
| 11. Girls are the victims of rumors if they have boyfriends | 0.600* | | 0.070 | | -0.110 | | 0.071 |
| 12. Boys tell girls they love them when they don't | 0.513* | | 0.059 | | 0.045 | | -0.124* |
| 13. A boy should have more than one girlfriend to gain experience | -0.238* | | 0.268* | | 0.407* | | 0.099 |
| 14. Adolescent girls should avoid boys because they trick them into having sex | 0.582* | | 0.006 | | -0.187* | | 0.071 |
| 15. Boys have girlfriends to show off to their friends | 0.536* | | 0.022 | | 0.134* | | -0.045 |
| 16. A girl should have more than one boyfriend to gain experience | -0.202* | | 0.287* | | 0.435* | | 0.140* |
| 17. Boys generally compete for the prettiest girls | 0.562* | | 0.281* | | 0.018 | | -0.043 |
| 18. A girl can have a boyfriend as long as she continues working well in school | 0.016 | | 0.879* | | -0.068 | | -0.034 |
| 20. A boy can have a girlfriend as long as he continues working well in school | 0.029 | | 0.900* | | -0.018 | | 0.011 |
| 21. Adolescent boys lose interest in a girl after they have sex with her | 0.364* | | 0.161* | | 0.048 | | -0.147* |
| 22. It’s normal for a girl to want a boyfriend at your age | 0.121* | | 0.489* | | 0.222* | | 0.041 |
| 23. Adolescent boys fool girls into having sex | 0.589* | | 0.012 | | 0.111 | | -0.063 |
| 24. It is ok for an adolescent girl to have sex as long as she avoids getting pregnant | -0.010 | | 0.410* | | 0.417* | | -0.009 |
| 25. In general, a girl should only have sex with someone she loves | 0.206* | | -0.021 | | 0.772* | | -0.013 |
| 27. It is ok for an adolescent boy to have sex as long as he avoids getting a girl pregnant | 0.005 | | 0.405* | | 0.390* | | -0.092 |
| 28. In general, a boy should only have sex with someone he loves | 0.147 | | -0.112 | | 0.835* | | -0.089 |
| 30. Girls should be proud of their bodies as they become women | 0.554* | | 0.002 | | -0.057 | | 0.068 |
| 40. It's important for boys to show they are tough | 0.060 | | -0.020 | | 0.147* | | 0.452* |
| 41. It is okay to tease a girl who acts like a boy | 0.024 | | 0.023 | | 0.000 | | 0.861* |
| 42. It is okay to tease a boy who acts like a girl | 0.007 | | -0.007 | | 0.013 | | 1.041* |
| Factor correlations | | | | | | | |
| F1 | | 1 | |  | |  |  |
| F2 | | 0.123* | | 1 | |  |  |
| F3 | | 0.110 | | 0.410* | | 1 |  |
| F4 | | 0.051 | | 0.131* | | 0.138* | 1 |

| **Number of factors** | **CFI**^α^ | **TLI**^β^ | **RMSEA**^∞^ **(90%CI)** | **SRMR**^ծ^ |
| --- | --- | --- | --- | --- |
| **EFA** |  |  |  |  |
| 1 | 0.65 | 0.63 | 0.16 (0.16-0.17) | 0.17 |
| 2 | 0.82 | 0.79 | 0.12 (0.12-0.13) | 0.12 |
| 3 | 0.94 | 0.92 | 0.07 (0.07-0.08) | 0.07 |
| 4 | 0.97 | 0.96 | 0.06 (0.05-0.06) | 0.06 |
| 5 | 0.98 | 0.97 | 0.05 (0.04-0.05) | 0.05 |
| **CFA** |  |  |  | WRMR |
| 4 | 0.91 | 0.89 | 0.06 (0.06-0.07) | 1.49 |
| **SEM** | 0.90 | 0.90 | 0.05 (0.04-0.06) | 1.34 |
| *Note: ^α^CFI(Comparative fit index) compares the fit of a target model to the fit of an independent, or null, model; ^β^TLI(Tucker-Lewis index ) is an incremental fit index, with a higher value indicating a better model fit; ^∞^RMSEA (root mean squared error of approximation) is a parsimony-adjusted index of the difference between observed covariance matrix per degree of freedom and the hypothesized covariance matrix; ^ծ^SRMR ( standardized root mean square residual ) gives the square-root of the difference between the residuals of the sample covariance matrix and the hypothesized model* | | | | |

**Table S3. Fit statistics for the exploratory factor analysis and confirmatory factor analysis for the gender norm scale items and the structural equation model, Kenya 2018**

**Table S4. Confirmatory factor analysis model for the gender norms scale among very young male adolescents in Korogocho slums, Kenya, 2018**

| **Items** | **Factors** |  |  |  |
| --- | --- | --- | --- | --- |
|  | **Sexual double standard** | **Heteronormative romantic relationships** | **Relational autonomy** | **Stereotypical views about toughness and weakness** |
| 1. A girl will lose interest in studying if she has a boyfriend | 0.427 |  |  |  |
| 3. Girls your age often get into "trouble" when they have boyfriends | 0.516 |  |  |  |
| 5. Boys have girlfriends for fun more than love | 0.510 |  |  |  |
| 7. Girls who have boyfriends are irresponsible | 0.435 |  |  |  |
| 9. Boys like girls who wear revealing clothes | 0.534 |  |  |  |
| 11. Girls are the victims of rumors if they have boyfriends | 0.590 |  |  |  |
| 12. Boys tell girls they love them when they don't | 0.537 |  |  |  |
| 14. Adolescent girls should avoid boys because they trick them into having sex | 0.525 |  |  |  |
| 15. Boys have girlfriends to show off to their friends | 0.580 |  |  |  |
| 17. Boys generally compete for the prettiest girls | 0.657 |  |  |  |
| 21. Adolescent boys lose interest in a girl after they have sex with her | 0.421 |  |  |  |
| 23. Adolescent boys fool girls into having sex | 0.613 |  |  |  |
| 30. Girls should be proud of their bodies as they become women | 0.525 |  |  |  |
| 6. It’s normal for a boy your age to want a girlfriend |  | 0.634 |  |  |
| 8. Boys should have girlfriends to discover love |  | 0.683 |  |  |
| 18. A girl can have a boyfriend as long as she continues working well in school |  | 0.783 |  |  |
| 20. A boy can have a girlfriend as long as he continues working well in school |  | 0.853 |  |  |
| 22. It’s normal for a girl to want a boyfriend at your age |  | 0.660 |  |  |
| 27. It is ok for an adolescent boy to have sex as long as he avoids getting a girl pregnant | | 0.673 |  |  |
| 4. A boy should be able to have a girlfriend if he wants to |  |  | 0.710 |  |
| 10A girl should be able to have a boyfriend if she wants to |  |  | 0.716 |  |
| 13. A boy should have more than one girlfriend to gain experience |  |  | 0.548 |  |
| 16. A girl should have more than one boyfriend to gain experience |  |  | 0.618 |  |
| 24. It is ok for an adolescent girl to have sex as long as she avoids getting pregnant |  |  | 0.713 |  |
| 25. In general, a girl should only have sex with someone she loves |  |  | 0.748 |  |
| 28. In general, a boy should only have sex with someone he loves |  |  | 0.699 |  |
| 40. It's important for boys to show they are tough |  |  |  | 0.526 |
| 41. It is okay to tease a girl who acts like a boy |  |  |  | 0.900 |
| 42. It is okay to tease a boy who acts like a girl |  |  |  | 1.001 |
| **Factor correlations** |  |  |  |  |
| Sexual double standard | 1 |  |  |  |
| Normative romantic relationships | 0.223 | 1 |  |  |
| Stereotypical views about toughness and weakness | 0.095 | 0.754 | 1 |  |
| Relational autonomy | 0.074 | 0.174 | 0.256 | 1 |
| Cronbach Alpha | 0.648 | 0.715 | 0.543 | 0.485 |

*Note: Data are standardized factor loadings and factor correlation*
